# Unmasking adrenoleukodystrophy in a cohort of cerebellar ataxia

**DOI:** 10.1371/journal.pone.0177296

**Published:** 2017-05-08

**Authors:** Ying-Hao Chen, Yi-Chung Lee, Yu-Shuen Tsai, Yuh-Cherng Guo, Cheng-Tsung Hsiao, Pei-Chien Tsai, Jin-An Huang, Yi-Chu Liao, Bing-Wen Soong

**Affiliations:** 1 Department of Neurology, Taipei Veterans General Hospital, Taipei, Taiwan; 2 Department of Neurology, School of Medicine, National Yang-Ming University, Taipei, Taiwan; 3 Brain Research Center, National Yang-Ming University, Taipei, Taiwan; 4 Center for Systems and Synthetic Biology, National Yang-Ming University, Taipei, Taiwan; 5 Institute of Clinical Medicine, National Yang-Ming University, Taipei, Taiwan; 6 Department of Neurology, China Medical University Hospital, Taichung, Taiwan; 7 School of Medicine, College of Medicine, China Medical University, Taichung, Taiwan; 8 Division of Neurology, Department of Internal Medicine, Taipei Veterans General Hospital Taoyuan Branch, Taoyuan, Taiwan; 9 Graduate Institute of Physiology, National Taiwan University College of Medicine, Taipei, Taiwan; 10 Neurological Institute, Taichung Veterans General Hospital, Taichung, Taiwan; Huashan Hospital Fudan University, CHINA

## Abstract

Adrenoleukodystrophy (ALD) is a rare and progressive neurogenetic disease that may manifest disparate symptoms. The present study aims at investigating the role of ataxic variant of ALD (AVALD) in patients with adult-onset cerebellar ataxia, as well as characterizing their clinical features that distinguish AVALD from other cerebellar ataxias. Mutations in the ATP binding cassette subfamily D member 1 gene (*ABCD1*) were ascertained in 516 unrelated patients with ataxia. The patients were categorized into three groups: molecularly unassigned hereditary ataxia (n = 118), sporadic ataxia with autonomic dysfunctions (n = 296), and sporadic ataxia without autonomic dysfunctions (n = 102). Brain MRIs were scrutinized for white matter hyperintensity (WMH) in the parieto-occipital lobes, frontal lobes, corticospinal tracts, pons, middle cerebellar peduncles and cerebellar hemispheres. Two *ABCD1* mutations (p.S108L and p.P623fs) previously linked to cerebral ALD and adrenomyeloneuropathy but not AVALD were identified. ALD accounts for 0.85% (1/118) of the patients with molecularly unassigned hereditary ataxia and 0.34% (1/296) of the patients with sporadic ataxia with autonomic dysfunctions. WMH in the corticospinal tracts and WMH in the cerebellar hemispheres were strongly associated with AVALD rather than other ataxias. To conclude, ALD accounts for approximately 0.39% (2/516) of adult-onset cerebellar ataxias. This study expands the mutational spectrum of AVALD and underscores the importance of considering ALD as a potential etiology of cerebellar ataxia.

## Introduction

Cerebellar ataxia comprises a heterogeneous group of diseases including hereditary ataxia, sporadic degenerative ataxia and acquired ataxia with exogenous etiologies, i.e. intoxication with alcohol or toxins and paraneoplastic syndrome [1, 2]. Making diagnosis in patients with a chronic progressive ataxia is clinically challenging since different ataxia diseases may have overlapping phenotypes and heterogeneous presentations [3]. Even after a comprehensive survey, genetic causes in 35–50% of autosomal dominant ataxia and 40–46% of autosomal recessive ataxia remain elusive [4, 5]. Hereditary diseases may also manifest as adult-onset sporadic ataxia. In fact, 2–22% of sporadic cases carry a genetic mutation, most frequently Friedreich’s ataxia or spinocerebellar ataxia (SCA) type 6 [6, 7].

X-linked adrenoleukodystrophy (ALD) is often overlooked in the differential diagnosis of adult-onset ataxia. ALD is caused by mutations in the ATP-binding cassette subfamily D member 1 gene (*ABCD1*) [8]. The clinical manifestations of ALD include a rapidly progressive childhood-, adolescence-, or adult-onset cerebral ALD, adrenomyeloneuropathy (AMN) presenting as a progressive paraplegia, and Addison’s disease without neurological deficits [9, 10]. The ataxic variant of ALD (AVALD) in which clinical phenotype resembles spinocerebellar degeneration was first reported in 1982 [11] and was later found to account for 1–2% of overall ALD [10]. To date, only a few AVALD cases have been reported [12] and the role of AVALD in patients with cerebellar ataxia remains unclear.

In the present study, we aimed to determine the frequency and spectrum of *ABCD1* mutations in a Taiwanese cohort of cerebellar ataxia, consisting of patients with hereditary ataxia and those with apparently sporadic ataxia. Clinical and brain MRI features of AVALD patients in this study and in the literature were analyzed to help distinguish AVALD from other cerebellar ataxias. Nowadays, whole exome sequencing and targeted gene panels including *ABCD1* become readily accessible in the clinical practice. The comprehensive description of AVALD features would be valuable to interpret the clinical significance of unclassified variants identified by high throughput sequencing technologies.

## Material and methods

### Participants

We retrospectively studied 516 patients with cerebellar ataxia who visited the Neurology Clinic of Taipei Veterans General Hospital between 1999 and 2015. Study participants included three groups: (1) hereditary cerebellar ataxia, (2) sporadic cerebellar ataxia with autonomic dysfunctions and (3) sporadic cerebellar ataxia without autonomic dysfunctions. Patients were categorized as hereditary ataxia if he/she has one or more affected family members either in at least two generations (autosomal dominant ataxia), in siblings (autosomal recessive ataxia), or with consanguinity in parents (autosomal recessive ataxia), or a disease onset before 25 years of age without any family history (autosomal recessive ataxia). For the 118 unrelated patients with hereditary ataxia, mutations responsible for SCA1, 2, 3, 6, 7, 8, 10, 12, 17, 19/22, 23, 26, 27, 28, 31, 35, 36, dentatorubral-pallidoluysian atrophy (DRPLA) and Friedreich’s ataxia had been excluded [13–15]. Mutations for SCA5, 11, 13, 14, 15/16, 38, 40, 41, 42, and 43 were not tested in our cohort because these subtypes were rarely identified in Asian populations.

Patients with a symptom onset later than 25 years of age and having no family history were categorized as sporadic ataxia (N = 398). Presence of any of the following manifestations, including postural hypotension, impotence, urinary retention, urinary incontinence and constipation, was suggestive of having autonomic dysfunctions. The presence of postural hypotension was defined by a drop of >20 mmHg in systolic blood pressure, >10 mm Hg in diastolic blood pressure or both during the tilting table test. SCA1, 2, 3, 6, 17 and Friedreich’s ataxia had also been excluded in these patients with sporadic ataxia. All participants were of Han Chinese descent and, prior to participating in the study, had given written informed consent that was approved by the Institutional Review Board of the Taipei Veterans General Hospital.

### MRI features

Brain magnetic resonance imaging (MRI) of the 516 patients were carefully reviewed. Axial MR brain images encompassing the entire cerebrum and cerebellum were acquired using a 1.5-T MR unit (Signa, GE Medical Systems, Milwaukee, WI, USA; Magnetom, Siemems, Erlangen, Germany). Atrophy in the brainstem and cerebellum as well as global atrophy were evaluated on T1-weighted images. White matter hyperintensity (WMH) on T2-weighted images or fluid attenuation inversion recovery (FLAIR) images was assessed in [16, 17]: (1) the parieto-occipital lobes and the splenium of corpus callosum, (2) the frontal lobes and the genu of corpus callosum, (3) corticospinal projection fibers (including internal capsules, cerebral peduncles, and basal pons), (4) middle cerebellar peduncles (MCPs), and (5) cerebellar hemispheres including dentate nuclei. In the pons, a cruciform hyperintensity referred as the “hot cross bun sign” (HCBS) and the preceding midline linear hyperintensity were also assessed [18, 19].

### Mutation detection and *in silico* prediction

Genomic DNA was isolated from the peripheral blood leukocytes following a standard protocol. The exons of *ABCD1* were amplified by PCR using intronic primers (S1 Table). Both the sense and antisense strands of all the amplicons were sequenced using the Big Dye 3.1 dideoxy terminator method with an ABI Prism 3700 Genetic Analyzer (Applied Biosystems, Foster City, CA). The amplicon sequences were compared against the published human *ABCD1* sequence (RefSeq accession number NM_000033.3) in the National Center for Biotechnology Information (NCBI) database (http://www.ncbi.nlm.nih.gov).

Polymorphism Phenotyping Program version 2 [20] and Mutation Taster algorithm [21] were used to *in silico* predict the biological impact of the identified variants of *ABCD1*. Phylogenetic conservation of the missense variant was estimated by aligning the amino acid sequences from several species (retrieved from the Entrez protein database of the NCBI) using ClustalX2.012 [22].

### Clinical evaluation of patients with AVALD

Clinical data, including the age at symptom onset (AO), disease duration, family history, autonomic dysfunctions, psychiatric symptoms (e.g. personality and mood changes), cognitive decline, alopecia, hyperpigmentation and visual impairment, were obtained from our ALD patients. Global cognitive performance was assessed using the Mini-Mental State Examination (MMSE) [23]. Motor and sensory nerve conduction studies (NCS) were conducted to evaluate the peripheral nervous system involvement. Plasma adrenocorticotropic hormone (ACTH) and cortisol concentrations were measured. Abnormal plasma levels of very long chain fatty acids (VLCFA) were defined as increased C26:0/C22:0 and C24:0/C22:0 ratios.

Among the 37 cases with AVALD in the literature [12, 17, 24–38], brain MRI information was available in 18 individuals. Clinical data, brain MRI features regarding the WMH in the corticospinal tracts and cerebellar hemispheres, plasma VLCFA levels and mutations in *ABCD1* were also obtained from the literature review.

## Results

### Identification of *ABCD1* mutations in our cohort of patients with ataxia

**Fig 1:**
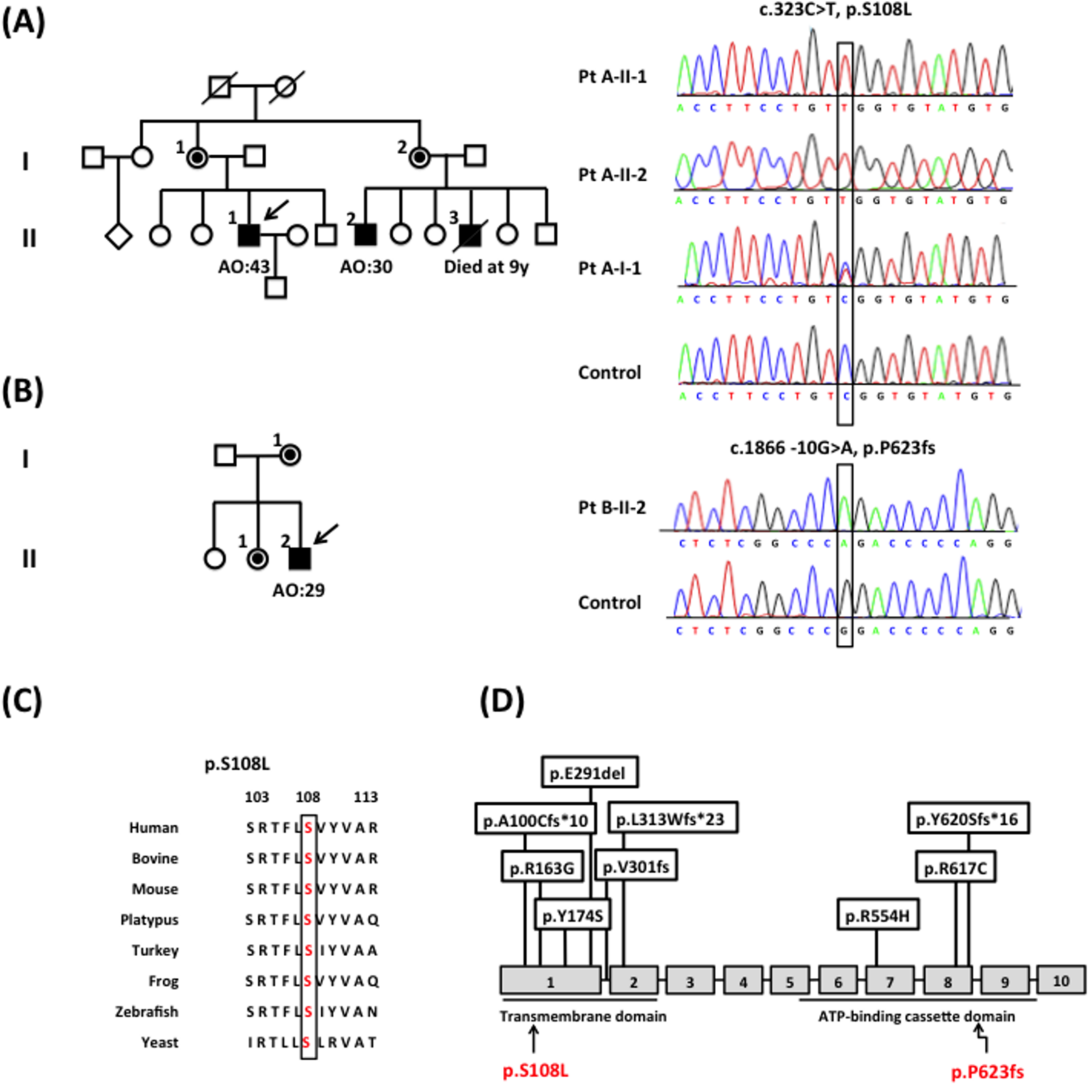
The *ABCD1* mutations identified in patients with ataxia. **(A-B)** The pedigrees and electropherograms of the patients with AVALD identified in the present study. Open symbol: unaffected; filled symbol: affected; symbol with a dot: asymptomatic heterozygotes; symbol with a diagonal line: deceased; square: males; circle: females; arrow: the proband. **(C)** The *ABCD1* p.S108L mutation occurs at an evolutionarily highly conserved residue, as shown by aligning the amino acid sequences of ATP-binding cassette sub-family D member 1 protein orthologs from various species. **(D)** The 11 mutations in *ABCD1* identified in patients with AVALD in the literature (9 mutations in the upper panel) and in the present study (2 mutations in the lower panel).

**Fig 2:**
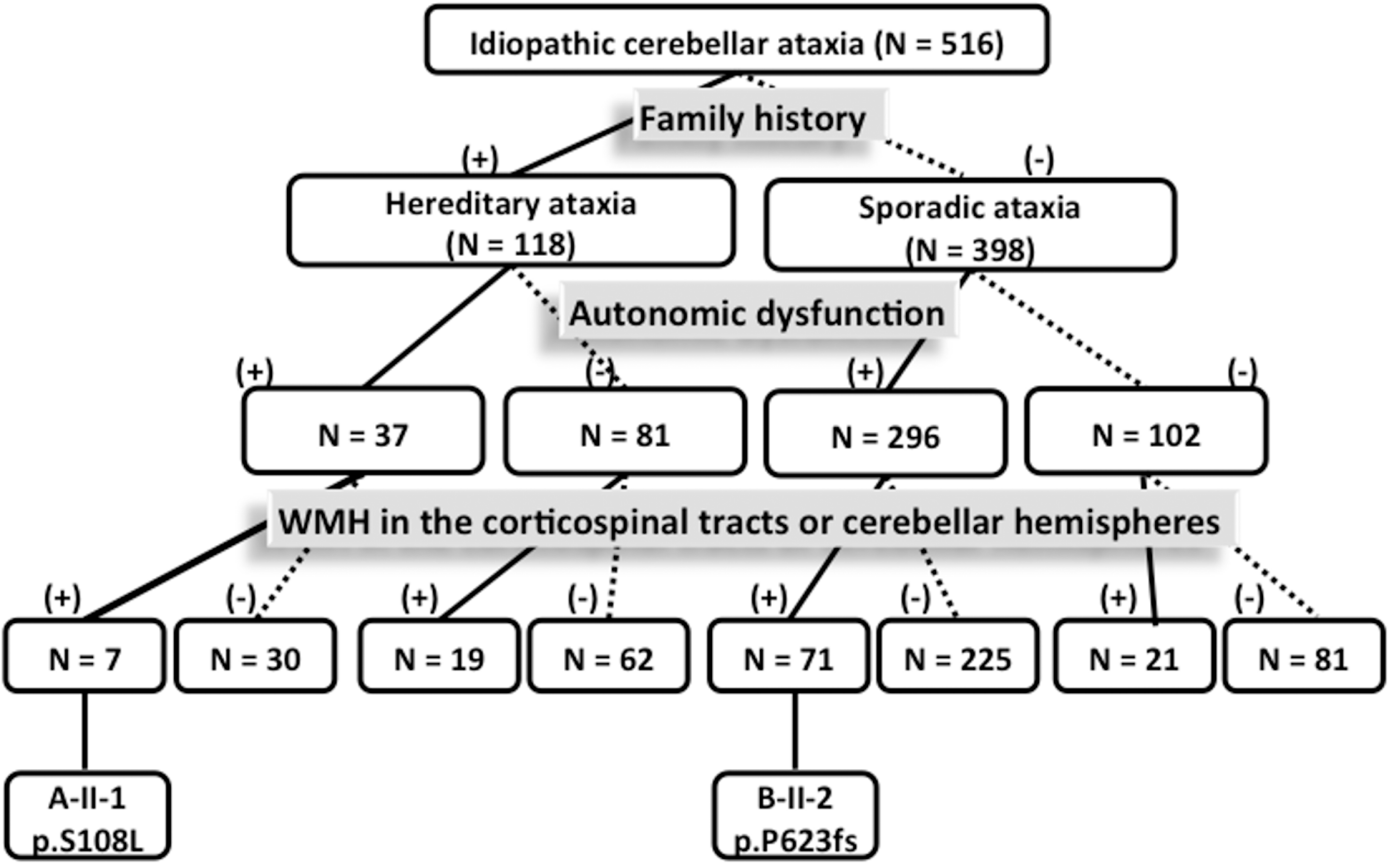
The algorithm of patient classification.

Among the 516 unrelated ataxic patients, one missense mutation (c.323C>T, p.S108L) and one splice site mutation (c.1866-10G>A, p.P623fs) in *ABCD1* were identified in one index patient each (Fig 1A and 1B). Patient A-II-1, who carried the p.S108L mutation, was previously diagnosed as hereditary cerebellar ataxia, while patient B-II-2 harboring the p.P623fs mutation was categorized into the group of sporadic ataxia with autonomic dysfunctions (Fig 2). In our cohorts, ALD accounted for 0.85% (1/118) of molecularly unassigned hereditary ataxia and 0.34% (1/296) of sporadic ataxia with autonomic dysfunctions or 0.25% (1/398) of all patients with sporadic ataxia.

The p.S108L mutation occurs at an evolutionarily highly conserved amino acid residue (Fig 1C), and the splice site mutation (c.1866-10G>A, p.P623fs) causes a frameshift of the coding sequence disrupting the ATP-binding cassette domain of *ABCD1*. In the literature, nine mutations in *ABCD1* had been identified in patients with AVALD (Fig 1D) [12, 26–29, 31, 32, 38]. Although the mutations p.P623fs and p.S108L had been previously reported in patients with ALD (http://www.x-ald.nl/), they manifested as cerebral ALD or AMN rather than ataxia (S2 Table) [39–42].

### Clinical features of the ataxic patients with *ABCD1* mutations

**Fig 3:**
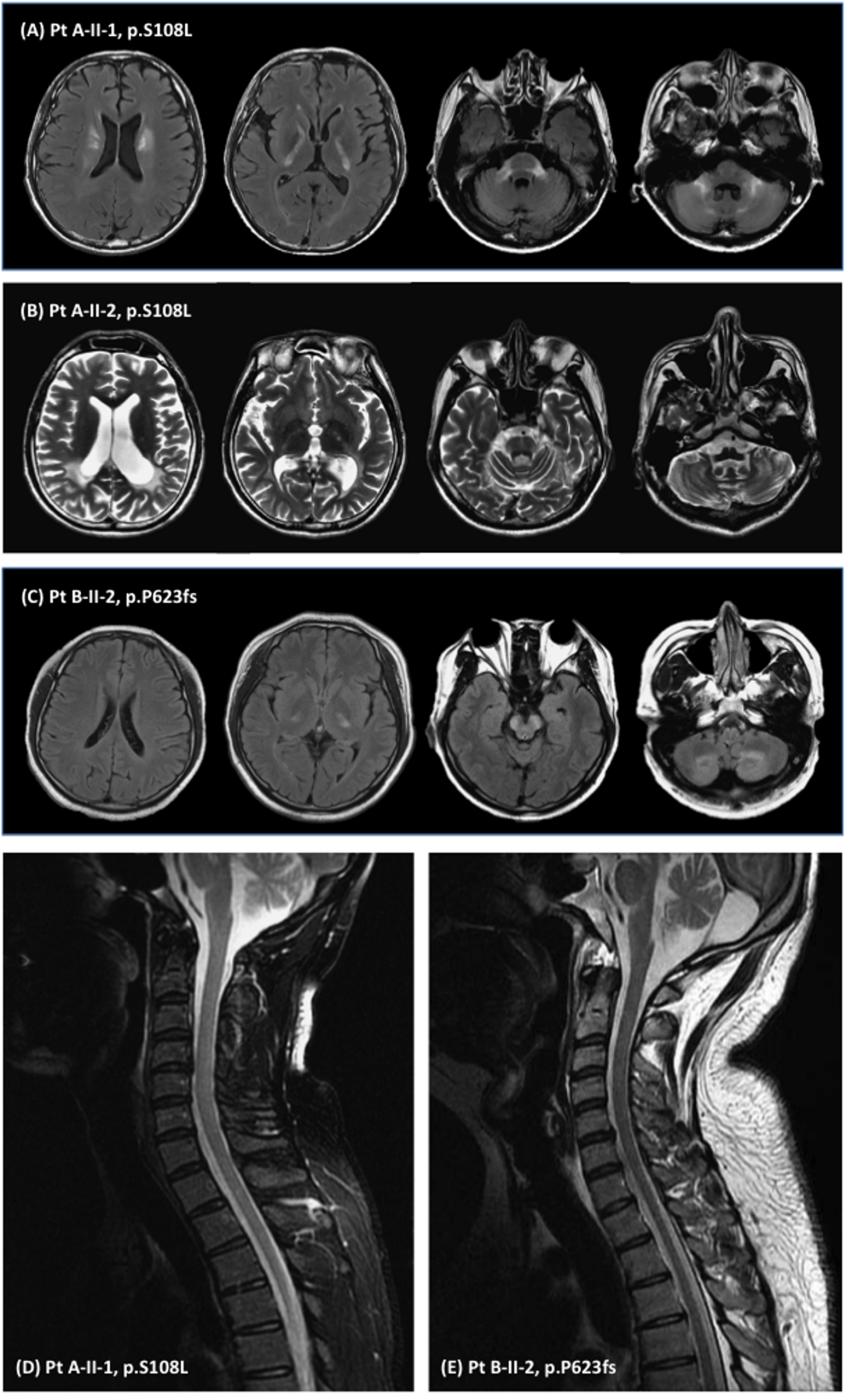
The brain and spinal cord magnetic resonance images of patients with *ABCD1* mutations. T2-weighted images or fluid attenuation inversion recovery (FLAIR) images to illustrate white matter hyperintensities (WMH) in **(A)** patient A-II-1, **(B)** patient A-II-2, and **(C)** patient B-II-2. Spinal cord MRI showed **(D)** normal findings in patient A-II-1 and **(E)** mild atrophy of the thoracic spinal cord in patient B-II-2.

Clinical and MRI features of the 2 AVALD patients are summarized in Table 1 and Fig 3.

**Table 1:** Characteristics of AVALD patients in the present study.

		A-II-1	B-II-2
Mutations in *ABCD1*		c.323C>T, p.S108L	c.1866-10G>A, p.P623fs
Demographic features	Sex	Male	Male
	AO/AE (y)	43/44	29/30
	Family history	Cousin	-
Autonomic dysfunction	Urinary/stool dysfunction	**+**	+
	Erectile dysfunction	**+**	-
	Postural hypotension	**+**	NA
Endocrine dysfunction	Adrenal insufficiency	ACTH:↑, cortisol: N	ACTH:↑, cortisol: N
	Hyperpigmentation	**+**	**+**
	Premature baldness	**+**	**-**
Psychiatric symptoms	Bipolar disorder	**+**	**-**
Neurological signs	Gait ataxia/limb dysmetria	**+/+**	**+/+**
	Tremor/rigidity	**-/-**	**-/-**
	Scanning/Spastic dysarthria	**+/-**	**+/-**
	Saccadic pursuit/Nystagmus	**+/-**	**+/-**
	Sensory deficit	-	-
	Limbs spasticity	-	-
	DTR/Babinski sign	Brisker/**+**	N/**+**
NCS		N	N
MMSE		27	NA
MRI findings	WMH in the parieto-occipital regions	**-**	**-**
	WMH in the frontal regions	**-**	**-**
	WMH in the cortico-spinal projection fibers	**+**	**+**
	WMH in the middle cerebellar peduncles	**+**	**-**
	WMH in cerebellar hemispheres	**+**	**+**
	Hot-cross bun sign in the pons	**+**	**-**
	Global brain atrophy	**-**	**-**
	Brainstem/cerebellar atrophy	**+**	**+**
Plasma levels of VLCFA	C26:0/C22:0 ratio C24:0/C22:0 ratio	0.0343 1.2365	0.0412 1.3792

**+**: presence; **-**: absence; ↑: elevated. *ABCD1*: ATP binding cassette subfamily D member 1 gene; ACTH: adrenocorticotropic hormone; AE: age at examination; AO: age at symptom onset; AVALD: ataxic variant of adrenoleukodystrophy, DTR: deep tendon reflexes; N: normal; NA: not available; MMSE: mini-mental state examination; NCS: nerve conduction studies; WMH: white matter hyperintensities in the T2WI/FLAIR images of brain magnetic resonance images; VLCFA: very long chain fatty acids (normal range: C26:0/C22:0 ratio = 0.0054–0.0235, C24:0/C22:0 ratio = 0.231–0.892)

Patient A-II-1, who had a past history of bipolar disorder, suffered from a progressive gait ataxia since age 43 years. Soon afterward, he developed postural hypotension, impotence, urinary incontinence, scanning speech, dysphagia, as well as impairment of cognitive functions and visual acuity. Neurological examination revealed a wide-based gait and clumsiness at rapid-alternative movements of the hands, saccadic pursuits, generalized brisk deep tendon reflexes, and extensor plantar responses. His muscle tone was normal without spasticity, and there was no sensory deficit. He had premature baldness and skin hyperpigmentation and was diagnosed to have adrenal insufficiency with a markedly elevated plasma ACTH level (1183 pg/ml; normal < 46) and a normal cortisol level (6.9 ug/dl; normal range: 3.7–19.4). Plasma levels of VLCFA were elevated (Table 1).

Patient A-II-1’s mother (A-I-1, Fig 1A) had a mild dysarthria without cognitive dysfunctions or gait difficulty. Patient A-II-2 had a depression and mild cognitive decline since age 30 and developed ataxic symptoms and autonomic dysfunctions since age 40. The other manifestations of patient A-II-2 included dysarthria, dysphagia, visual disturbance and autonomic dysfunctions. Patient A-II-3 had also suffered from a visual impairment and died at age 9. Both patients (A-II-1, A-II-2) are hemizygous for c.323C>T (p.S108L) *ABCD1* mutation and the mother (A-I-1) is a heterozygote for the mutation. Brain MRI of A-II-1 and A-II-2 demonstrated WMH in the corticospinal projection fibers extending from the corona radiate to the posterior limbs of the internal capsules (Fig 3A and 3B). There were also WMH in MCPs, cerebellar hemispheres, dentate nuclei, as well as HCBS in the pons. Cervical cord MRI of patient A-II-1 did not reveal any abnormality (Fig 3D).

Patient B-II-2 presented with a progressive dysarthria and gait disturbance since age 29 years. His clinical manifestations resembled those of patient A-II-1 (Table 1) and had a poor academic performance at school since adolescence. Besides, he also had a skin hyperpigmentation, adrenal insufficiency, and elevated plasma VLCFA levels (Table 1). He was found to carry a c.1866-10G>A (p.P623fs) mutation in *ABCD1*. Although his mother (B-I-1) and one (B-II-1) of his elder sisters were later found to carry the heterozygous c.1866-10G>A (p.P623fs) mutation, they had remained asymptomatic. Brain MRI of the patient B-II-2 featured WMH in the posterior limbs of the internal capsules, cerebral peduncles, and cerebellar dentate nuclei (Fig 3C). Spinal cord MRI revealed a mild atrophy of the thoracic spinal cord (Fig 3E).

### Clues to identify ALD in patients with ataxia

We were wondering if any clinical or MRI features could serve to heighten the suspicion of ALD in patients with ataxia. The clinical and image characteristics were compared between patients with AVALD and other ataxia patients without *ABCD1* mutations (Table 2). Among all the clinical and image features, WMH in the corticospinal tracts and WMH in the cerebellar hemispheres were much more prevalent in AVALD (100% and 100%, respectively) than in other ataxias (13.6% and 10.1%, respectively). The two AVALD patients had WMH in both the corticospinal tracts and cerebellum hemispheres. For patients with other etiologies of ataxia in this study, only one fifth of them had WMH either in the corticospinal tracts or in the cerebellar hemispheres and they rarely had WMH in both regions (Table 2).

**Table 2:** Comparison between the patients with AVALD and the ataxic patients with other etiologies.

Mean ± SD or N (%)	Group 1 (N = 117)	Group 2 (N = 295)	Group 3 (N = 102)	Group 1+2+3 (N = 514)	AVALD (N = 2)
Male	56 (47.9%)	158 (53.6%)	49 (48.0%)	263 (51.2%)	2 (100%)
Family history	83 (70.9%)	0 (0.0%)	0 (0.0%)	83 (16.1%)	1 (50.0%)
AO (y)	37.4 ± 21.8	55.8 ± 8.5	52.3 ± 13.4	50.9 ± 15.5	36.0 ± 9.9
Disease duration (y)	7.96 ± 8.80	2.51 ± 2.20	3.38 ± 4.34	3.92 ± 5.38	1.00 ± 0.0
Autonomic dysfunction	36 (30.8%)	295 (100%)	0 (0.0%)	331 (64.4%)	2 (100%)
WMH in the parieto-occipital lobes	28 (23.9%)	93 (31.5%)	17 (16.7%)	138 (26.8%)	0 (0.0%)
WMH in the frontal lobes	14 (12.0%)	30 (10.2%)	7 (6.9%)	51 (9.9%)	0 (0.0%)
WMH in the corticospinal projection fibers (A)	21 (17.9%)	33 (11.2%)	16 (15.7%)	70 (13.6%)	2 (100%)
WMH in the cerebellar hemispheres (B)	5 (4.3%)	41 (13.9%)	6 (5.9%)	52 (10.1%)	2 (100%)
(A) or (B)	25 (21.4%)	70 (23.7%)	21 (20.6%)	116 (22.6%)	2 (100%)
(A) and (B)	1 (0.9%)	4 (1.4%)	1 (1.0%)	6 (1.2%)	2 (100%)
WMH in the middle cerebellar peduncles	16 (13.7%)	147 (49.8%)	17 (16.7%)	180 (35%)	1 (50.0%)
Hot-cross bun sign in the pons	21 (17.9%)	222 (75.3%)	29 (28.4%)	272 (52.9%)	1 (50.0%)
Global brain atrophy	23 (19.7%)	77 (26.1%)	28 (27.5%)	128 (24.9%)	0 (0.0%)
Brainstem/cerebellar atrophy	84 (71.8%)	271 (91.9%)	75 (73.5%)	430 (83.7%)	2 (100%)

AVALD: ataxic variant of adrenoleukodystrophy; AO: age at symptom onset; Group 1: hereditary cerebellar ataxia; Group 2: sporadic ataxia with autonomic dysfunction; Group 3: sporadic ataxia without autonomic dysfunction; WMH: white matter hyperintensities in the T2WI/FLAIR images of brain magnetic resonance images

Brain MRI features were available in 18 cases of AVALD in the literature (Table 3). Among those 18 cases, the diagnosis of ALD was ascertained by elevated plasma levels of VLCFA in 17 and the presence of *ABCD1* mutations in 9. All of them are males. The average AO was 30.4 ± 7.8 years (20–47). A positive family history was reported in only 53.3% of the patients, and autonomic dysfunctions were found in a half of them. WMH in the corticospinal tracts and cerebellum were found in 72.2% and 70.6%, respectively. Of note is that there was a high concordance rate for the presence of WMH in these two regions (76.5% in literature cases and 100% in the present study). Among the 18 literature cases of AVALD, up to 82.4% fulfilled at least one of the two diagnostic hints (the presence of WMH in the corticospinal tracts or cerebellar hemispheres), supporting the feasibility of this “red flag” to distinguish AVALD from other ataxic patients. Approximately 58.8% of them had WMH in both the corticospinal tracts and cerebellar hemispheres.

**Table 3:** Characteristics of patients with AVALD in the literature.

Patient /Reference	Ethnicity	Sex	AO (y)	Autonomic dysfunction	FHx	WMH		VLCFA	*ABCD1* mutation	Diagnostic hints	
						CST	Cerebellum			either	both
1 [24]	Korean	M	37	+	+	+	+	+	-	T	T
2 [17]	Japanese	M	26	+	+	+	+	+	-	T	T
3 [25]	Indian	M	26	+	NA	+	+	+	-	T	T
4 [26]	Taiwanese	M	28	+	+	+	-	+	c.1859delA, p.Tyr620Serfs*16	T	F
5 [27]	Korean	M	35	+	+	-	+	+	c.277_296dup20, p.Ala100Cysfs*10	T	F
6 [28]	Japanese	M	47	+	+	+	+	+	c.871_873delGAG, p.Glu291del	T	T
7 [29]	Korean	M	36	+	+	+	-	+	c.901-1G>A, p.Val301fs	T	F
8 [30]	Japanese	M	20	-	NA	+	+	+	-	T	T
9 [31]	Korean	M	36	-	-	+	+	+	c.521A>T, p.Tyr174Ser	T	T
10 [32]	Caucasian	M	37	+	+	-	-	+	c.937delC, p.Leu313Trpfs*23	F	F
11 [33]	Japanese	M	28.5	-	NA	+	+	+	-	T	T
12 [34]	Japanese	M	21	-	-	+	+	+	-	T	T
13 [35]	Japanese	M	29	-	-	+	+	+	-	T	T
14 [36]	American	M	41	-	+	+	+	+	-	T	T
15 [37]	NA	M	22.5	-	-	-	+	+	-	T	F
16 [38]	NA	M	21	-	-	-	-	+	c.1849C>T, p.Arg617Cys	F	F
17 [12]	Caucasian	M	34	+	-	-	-	NA	c.487C>G, p.Arg163Gly	F	F
18 [29]	Korean	M	23	-	-	+	NA	+	c.1661G>A, p.Arg554His	NA	NA
All		M	30.4 ± 7.8	50% (9/18)	53.3% (8/15)	72.2% (13/18)	70.6% (12/17)	100% (17/17)		82.4% (14/17)	58.8% (10/17)

T: true; F = false; +: presence; -: absence; *ABCD1*: ATP binding cassette subfamily D member 1 gene; AO: age at symptom onset; AVALD: ataxic variant of adrenoleukodystorphy; CST: corticospinal tracts; Diagnostic hints: presence of WMH in the corticospinal tracts or in the cerebellar hemisphere; FHx: family history; M: male; NA: not available; WMH: white matter hyperintensities in the T2WI/FLAIR images of brain magnetic resonance images; VLCFA: elevated plasma very long chain fatty acids levels

## Discussion

The present study highlights the importance of being aware of ALD in patients with cerebellar ataxia. The two *ABCD1* mutations (p.S108L and p.P623fs), previously reported to be linked to cerebral ALD and AMN [39–42], were reported for the first time in patients predominantly manifesting with cerebellar ataxia. It indicates that *ABCD1* mutations have a notably heterogeneous manifestations. The characteristic features of AVALD include a slowly progressive ataxia with mild limb dysmetria, scanning dysarthria, saccadic pursuits, accompanied by autonomic dysfunctions. Psychiatric symptoms, visual impairment and adrenal insufficiency were also frequently observed. There was no Parkinsonism feature (such as tremor or rigidity), spasticity or sensory deficits. Presence of WMH in the corticospinal tracts and cerebellar hemispheres could be red flags to suggest the possibility of AVALD in patients with ataxia. In addition, male gender, family history of affected maternal relatives, autonomic dysfunctions, psychiatric symptoms, skin hyperpigmentation, and adrenal insufficiency are all useful clues to heighten the clinical suspicion of ALD.

AVALD, once regarded as a rare variant and accounting for only 1–2% of ALD [10], might have been underestimated, at least in the Asians [43]. In a Japanese cohort with 145 ALD patients, 13 patients (8.4%) manifested with olivopontocerebellar form [43]. Similarly, the cerebellar subtype of MSA (MSA-C) are more frequently observed in Japan and Taiwan than in Europe or the United States [44]. Some environmental or genetic factors might contribute to the higher prevalence of AVALD in the Asians. In our cohort, ALD was found in 0.85% of patients with molecularly unassigned hereditary ataxia and 0.34% of patients with sporadic ataxia with autonomic dysfunctions. Since 22% of patients with hereditary ataxia in Taiwan have an unassigned genetic diagnosis, AVALD appears to account for a lower percentage (0.19%) of all hereditary ataxias in comparison to that of SCA3 (47%), SCA2 (11%), SCA6 (11%), SCA1 (5%), SCA17 (2.7%) in our population [45]. Of note, AVALD could masquerade as recessive spinocerebellar ataxia or MSA-C, but it is frequently overlooked in the differential diagnosis of hereditary ataxia or adult-onset sporadic ataxia [1, 46]. Only two papers had suggested considering ALD in X-linked ataxia, especially in those with an AO younger than 50 [4, 47].

AO might not be a reliable clue for AVALD. In the literature, the AO for AVALD ranged from 20 to 54 years [12], overlapping with that of most hereditary ataxia ranging between twenties and forties. An AO beyond 55 might be a hint to favor MSA-C rather than AVALD, in patients with cerebellar ataxia and autonomic dysfunctions [6, 19]. In our cohort, WMH in both the corticospinal tracts and cerebellar hemispheres was observed in AVALD patients (100% and 100%) but rarely in patients of sporadic ataxia with autonomic dysfunctions (11.2% and 13.9%) (Table 2). HCBS, the characteristic features of MSA-C [48], was common in both patient groups (75.3% and 50% in sporadic ataxia with autonomic dysfunctions and AVALD, respectively). In rare instances, as described by Ogaki et al. and Vianello et al., the MRI of AVALD patients could only manifest diffuse atrophy without any WMH in the brainstem or cerebellum [12, 38]. In such cases, clinical features, such as endocrinological abnormality, psychiatric symptoms, and autonomic dysfunctions, are important clues.

The results from this study suggest that WMH in the cerebellum or corticospinal tracts is a distinguishing feature to identify ALD in patients with ataxia. However, a large, prospective cohort of AVALD is needed to validate such association. In concert with our findings, a 3-year annual MRI follow-up study demonstrated demyelination changes in the dentatorubothalamic tracts and corticospinal tracts in a patient with AVALD [33]. Another study with an autopsied case of AVALD indicated that these cerebellar white matter lesions reflect underlying pathology of myelin rarefaction and perivascular infiltrations of macrophages [12], a consequence of toxic accumulation of VLCFA in the oligodendrocytes. Of note, the presence of WMH in the cerebellar hemispheres or corticospinal tracts is not specific to AVALD. Other neurogenetic disorders, such as Fragile X-associated tremor/ataxic syndrome, MSA-C, Wilson’s disease, mitochondrial recessive ataxia syndrome with mutations in *POLG*, cerebrotendinous xanthomatosis, Krabbe disease, and peroxisomal disorders may also manifest with cerebellar ataxia accompanied by WMH in the cerebellum [1, 49, 50].

A negative family history would not exclude the possibility of ALD. Around 50% of women carrying a heterozygous *ABCD1* mutation may develop AMN-like symptoms after age 40, and less than 2% of them may have a cerebral involvement [10]. A negative family history or an apparently recessive inheritance but lack of a male-to-male transmission should raise the possibility of X-linked ataxia [4]. Another reason that family history is not entirely helpful in diagnosis of AVALD is the high phenotypic variation of *ABCD1* mutations. It has been well known that there is poor genotype-phenotype correlation in individuals with *ABCD1* mutations and the entire ALD spectrums could be observed within a pedigree with the same mutation [51].

In summary, AVALD accounts for approximately 0.39% (2/516) of cerebellar ataxias. This study expands the mutational spectrum of AVALD and stresses the importance of considering ALD as a potential etiology of cerebellar ataxia, especially in patients with autonomic dysfunctions. The presence of WMH in the corticospinal tracts or cerebellar hemispheres are important hints to distinguish ALD from other ataxias, especially in the scenario of male gender, family history of affected maternal relatives, autonomic dysfunctions, psychiatric symptoms, skin hyperpigmentation, premature baldness and adrenal insufficiency.

## Supporting information

S1 Table Primers used for sequencing *ABCD1*. (DOCX)

S2 Table Bioinformatics predictions of pathogenicity of the *ABCD1* mutations identified in this study. (DOCX)
