# Supplementary material for: Unmasking adrenoleukodystrophy in a cohort of cerebellar ataxia
Source: PLoS One. 2017 May 8;12(5):e0177296. doi: 10.1371/journal.pone.0177296 (PMC5421786; doi:10.1371/journal.pone.0177296)
Supplement: S1 Table — (DOCX) [file pone.0177296.s001.docx]

**S1 Table. Primers used for sequencing *ABCD1***

| Exon | Forward primer | Reverse primer |
| --- | --- | --- |
| Exon 1-1 | GTCAGAGCAACAATCCTTC | CCCGTCCATGTTGCTGAC |
| Exon 1-2 | ACTGGCCCTGTCGTTCCG | CCCACCGCTCACGGCTGC |
| Exon 2 | TGGGAGACCCTGACCATCG | CTCAGCACCCAGCGGTATG |
| Exon 3 | AAGAGCCTCGCCTTTCTCTC | CTGTGTAGCCAGCCAGCTC |
| Exon 4 | CATCCTTGCCATGCTTCTCT | CATGGAGGTCCCTGAGTGAG |
| Exon 5 | CAGAATGCAGAGGGGGTCG | CCTGCCACGTACATCTAGGG |
| Exon 6-7 | GAGCCTCTCAAGGCTGGTC | TGGTGTTGGTCCTCCCTG |
| Exon 8-10 | GTCACAGCTAGCTCATTCCCG | CAGGGGCCGGGGACGGTTGT |

The PCR reaction was performed using 100 ng DNA, 1X reaction buffer, 10 mM dNTP, 10 uM each primer and 1.25 u TAKARA Taq (TAKARA, R001A). The PCR condition was 95℃ for 5 min, (95℃ for 30s, 60℃ for 30s, 72℃ for 1 min) x 30 cycles, 72℃ for 10 min.
