# Supplementary material for: Unmasking adrenoleukodystrophy in a cohort of cerebellar ataxia
Source: PLoS One. 2017 May 8;12(5):e0177296. doi: 10.1371/journal.pone.0177296 (PMC5421786; doi:10.1371/journal.pone.0177296)
Supplement: S2 Table — (DOCX) [file pone.0177296.s002.docx]

**S2 Table. Bioinformatics predictions of pathogenicity of the *ABCD1* mutations identified in this study**

| Mutation | Protein | Location | Domain | Pathogenicity prediction | | Literature report |
| --- | --- | --- | --- | --- | --- | --- |
| Transcript |  |  |  | PolyPhen-2 | Mutation Taster |  |
| c.323C>T | p.S108L | Exon 1 | transmembrane domain | probably damaging | disease causing | 2 childhood cerebral ALD,  2 AMN [[1-3](#_ENREF_1)] |
| c.1866-10G>A | p.P623fs | IVS 8 | ATP-binding cassette | NA | NA | 1 childhood cerebral ALD,  1 adolescence cerebral ALD [[4](#_ENREF_4)] |

ALD: adrenoleukodystrophy; AMN: adrenomyeloneuropathy; IVS: intervening sequence; Polyphen-2 : Polymorphism Phenotyping Program version 2 [[5](#_ENREF_5)] (http://genetics.bwh.harvard.edu/pph2/); Mutation Taster algorithm[[6](#_ENREF_6)] (<http://www.mutationtaster.org/>)

**Reference**

1. Asheuer M, Bieche I, Laurendeau I, Moser A, Hainque B, Vidaud M, et al. Decreased expression of ABCD4 and BG1 genes early in the pathogenesis of X-linked adrenoleukodystrophy. Hum Mol Genet. 2005;14(10):1293-303. doi: 10.1093/hmg/ddi140. PubMed PMID: 15800013.

2. Matsukawa T, Asheuer M, Takahashi Y, Goto J, Suzuki Y, Shimozawa N, et al. Identification of novel SNPs of ABCD1, ABCD2, ABCD3, and ABCD4 genes in patients with X-linked adrenoleukodystrophy (ALD) based on comprehensive resequencing and association studies with ALD phenotypes. Neurogenetics. 2011;12(1):41-50. doi: 10.1007/s10048-010-0253-6. PubMed PMID: 20661612; PubMed Central PMCID: PMC3029816.

3. Perusi C, Gomez-Lira M, Mottes M, Pignatti PF, Bertini E, Cappa M, et al. Two novel missense mutations causing adrenoleukodystrophy in Italian patients. Mol Cell Probes. 1999;13(3):179-82. doi: 10.1006/mcpr.1999.0232. PubMed PMID: 10369742.

4. Kumar N, Taneja KK, Kalra V, Behari M, Aneja S, Bansal SK. Genomic profiling identifies novel mutations and SNPs in ABCD1 gene: a molecular, biochemical and clinical analysis of X-ALD cases in India. PLoS One. 2011;6(9):e25094. doi: 10.1371/journal.pone.0025094. PubMed PMID: 21966424; PubMed Central PMCID: PMC3178599.

5. Adzhubei IA, Schmidt S, Peshkin L, Ramensky VE, Gerasimova A, Bork P, et al. A method and server for predicting damaging missense mutations. Nat Methods. 2010;7(4):248-9. doi: 10.1038/nmeth0410-248. PubMed PMID: 20354512; PubMed Central PMCID: PMC2855889.

6. Schwarz JM, Cooper DN, Schuelke M, Seelow D. MutationTaster2: mutation prediction for the deep-sequencing age. Nat Methods. 2014;11(4):361-2. doi: 10.1038/nmeth.2890. PubMed PMID: 24681721.
